# Safety and Immunogenicity of Boosting BCG Vaccinated Subjects with BCG: Comparison with Boosting with a New TB Vaccine, MVA85A

**DOI:** 10.1371/journal.pone.0005934

**Published:** 2009-06-16

**Authors:** Kathryn T. Whelan, Ansar A. Pathan, Clare R. Sander, Helen A. Fletcher, Ian Poulton, Nicola C. Alder, Adrian V. S. Hill, Helen McShane

**Affiliations:** 1 Jenner Institute, University of Oxford, Churchill Hospital, Oxford, United Kingdom; 2 Centre for Clinical Vaccinology and Tropical Medicine, Churchill Hospital, Oxford, United Kingdom; 3 Centre for Statistics in Medicine, University of Oxford, Oxford, United Kingdom; University of Sao Paulo, Brazil

## Abstract

**Objectives:**

To investigate the safety and immunogenicity of a booster BCG vaccination delivered intradermally in healthy, BCG vaccinated subjects and to compare with a previous clinical trial where BCG vaccinated subjects were boosted with a new TB vaccine, MVA85A.

**Design:**

Phase I open label observational trial, in the UK. Healthy, HIV-negative, BCG vaccinated adults were recruited and vaccinated with BCG. The primary outcome was safety; the secondary outcome was cellular immune responses to antigen 85, overlapping peptides of antigen 85A and tuberculin purified protein derivative (PPD) detected by *ex vivo* interferon-gamma (IFN-γ) ELISpot assay and flow cytometry.

**Results and Conclusions:**

BCG revaccination (BCG-BCG) was well tolerated, and boosting of pre-existing PPD-specific T cell responses was observed. However, when these results were compared with data from a previous clinical trial, where BCG was boosted with MVA85A (BCG-MVA85A), MVA85A induced significantly higher levels (>2-fold) of antigen 85-specific CD4+ T cells (both antigen and peptide pool responses) than boosting with BCG, up to 52 weeks post-vaccination (p = 0.009). To identify antigen 85A-specific CD8+ T cells that were not detectable by *ex vivo* ELISpot and flow cytometry, dendritic cells (DC) were used to amplify CD8+ T cells from PBMC samples. We observed low, but detectable levels of antigen 85A-specific CD8+ T cells producing IFNγ (1.5% of total CD8 population) in the BCG primed subjects after BCG boosting in 1 (20%) of 5 subjects. In contrast, in BCG-MVA85A vaccinated subjects, high levels of antigen 85A-specific CD8+ T cells (up to 14% total CD8 population) were observed after boosting with MVA85A, in 4 (50%) of 8 subjects evaluated.

In conclusion, revaccination with BCG resulted in modest boosting of pre-existing immune responses to PPD and antigen 85, but vaccination with BCG-MVA85A induced a significantly higher response to antigen 85 and generated a higher frequency of antigen 85A-specific CD8+ T cells.

**Trial Registration:**

ClinicalTrials.gov NCT00654316 NCT00427830

## Introduction

It is estimated that one third of the world's population is infected with *Mycobacterium tuberculosis* (*M. tb.*) [1]. In most infected individuals the host response contains this infection, or may result in pathogen clearance. However, the global burden of disease remains a significant problem. In 2007, there were estimated to be 9.2 million new cases of tuberculosis (TB) and in 2006, there were 0.5 million cases of multi-drug resistant TB [1]. The only available vaccine against TB, *Mycobacterium bovis* Bacille Calmette-Guerin (BCG), has variable efficacy. When BCG is administered at birth, it confers consistent and reliable protection against disseminated disease in childhood in the developing world [2], [3]. However, BCG fails to protect against pulmonary disease in these regions [4]. Any improved vaccine regime against TB should include BCG, administered in infancy, in order to retain the protective effects against severe childhood disease [5], [6], [7].

Revaccination with BCG in adolescence has been routine practice in many countries throughout the world, with evidence to suggest that it confers improved protection against diseases such as TB meningitis [2] and leprosy [8]. In a large clinical trial of BCG revaccination with over 200,000 children in Brazil, no enhanced protective effect of boosting with BCG against pulmonary disease was observed [9]. In light of this and other studies, revaccination is not recommended by the WHO [10].

The immunological markers of protection against TB have not yet been defined. However, immunity to *M.tb*. is dependent on the generation of a Th1-type cellular immune response. Secretion of IFNγ is central to the activation of *M.tb*-infected macrophages and the measurement of IFNγ release from antigen-specific T cells provides the best available immunological correlate of protection against TB [11], [12], [13]. TB is the commonest HIV-related disease and the most frequent cause of mortality [1]. The annual risk of developing TB disease from reactivation of latent *M.tb* infection in those co-infected with HIV is 5–15% annually, compared to a 5–10% lifetime risk in immunocompetant individuals [14], [15]. In addition to the importance of CD4+ T cells in protective immunity, CD8+ T cells are also important in the control of *M.tb* infection. CD8+ T cells may be of greater importance during the latent stages of *M.tb* infection, by maintaining control of slowly replicating bacteria, rather than in acute infection [16], [17]. Improved induction, activation, or functionality of TB-specific CD8+ T cells may enhance protective immunity against TB [18].

We have developed a new vaccine, Modified Vaccinia Ankara expressing antigen 85A (MVA85A) using a recombinant viral vector system that is designed to induce and amplify the cellular immune response. This vaccine has been developed as a booster vaccine for BCG. Antigen 85A is an enzyme (mycolyl-transferase) involved in cell wall biosynthesis, is highly conserved in all mycobacterial species, and is present in all strains of BCG [19], [20]. Antigen 85A is immunodominant in animal and human studies [21], [22], [23], and immunization with antigen 85A conferred significant protection against *M.tb* challenge [21], [22], [23]. Boosting BCG with MVA85A induces high levels of antigen specific IFNγ secreting CD4+ and CD8+ cells and enhanced protection against *M.tb* challenge when administered intranasally to mice. In larger animals enhanced protection was observed when MVA85A was administered intradermally to boost BCG in non human primates (Verreck F et al, submitted) and also to cattle where protection against *M. bovis* challenge was enhanced (Vordermeier M et al, submitted). In a series of clinical trials we have shown that MVA85A is effective at inducing high levels of antigen specific CD4+ T cells when given alone or as a boost to BCG induced immune responses [24]. However, detecting TB-specific CD8+ T cells in peripheral blood mononuclear cells (PBMC) samples from these clinical trials proved to be difficult. Using *ex vivo* ELISpot assays and PBMC based flow cytometry, no antigen specific CD8+ T cell responses were detected either before or after MVA85A vaccination in any of the studies to date. In other work, dendritic cells (DC) have been employed as professional antigen presenting cells, to increase the sensitivity of CD8+ T cell detection. This is due to the high levels of MHC I and MHC II, co-stimulatory molecules and relevant cytokine profile expressed by mature DC which facilitates expansion of antigen-specific T cell population that otherwise may not be detected [25], [26], [27], [28].

The aim of the clinical trial presented here was to investigate the safety and immunogenicity of boosting BCG vaccinated subjects with BCG, and to compare these results with data from previous trials where BCG vaccinated subjects had been boosted with MVA85A [24]. In addition, the particular immunological focus was to establish a methodology for detecting antigen specific CD8+ T cell responses in these subjects.

## Methods

**Figure 1:**
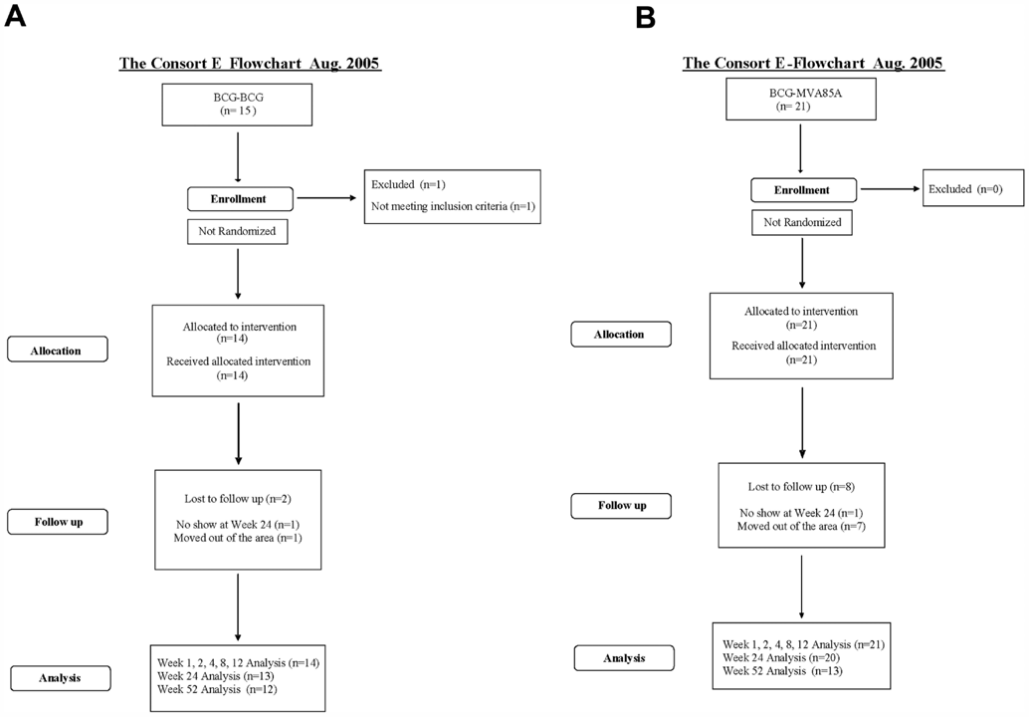
Consort Flowcharts for (a) BCG-BCG and (b) BCG-MVA85A Vaccination Regimens After 52 Weeks Post-Vaccination.

Consort flowcharts for each of the two trials discussed here are presented in Figure 1. The protocol for this trial and supporting CONSORT checklist are available as supporting information; see Checklist S1 and Protocol S1.

### Participants

Subjects were recruited for both clinical trials under protocols approved by the Oxfordshire Research Ethics Committee and enrolled only after obtaining written informed consent. Follow up was for 24 weeks; however protocols were amended during the trial so that subjects still living in the area were invited for a week 52 assessment. The trials reported here were open label observational trials.

Subjects enrolled in the clinical trials were all healthy and had previously been vaccinated with BCG. This was confirmed visually by the presence of a BCG scar. They were all seronegative for HIV, HBV and HCV at screening, and routine laboratory haematology and biochemistry were performed prior to vaccination. All values were within normal limits. All subjects had a Heaf test and anyone with a Heaf test score of greater than Grade 2 was excluded. All subjects were negative for the *M.tb* -specific antigens ESAT6 and CFP10, as determined by an *ex vivo* ELISpot assay.

There were 14 subjects eligible for enrolment into the BCG-BCG trial with a median time interval of 12 years from their first BCG vaccination. The median age of subjects was 28 years (range 19 to 51 years). Subjects in the BCG-MVA85A trial were from a previously reported study (n = 17) [24], with an additional 4 subjects subsequently recruited into the BCG-MVA85A group. All 21 subjects were used in the analysis for this paper. Similar inclusion/exclusion criteria were applied to subjects recruited to the BCG-BCG and BCG-MVA85A studies [24]. The median interval period between BCG vaccination and boosting with MVA85A was 18 years in this group, and the median age of subjects was 31 years (range 22 to 54 years). Demographic information on all the subjects is summarised in Table 1.

**Table 1:** Demographic Details of Subjects in BCG-BCG and BCG-MVA85A Trials.

	BCG–BCG (n = 14)	BCG–MVA85A (n = 21)
**Gender**	Male 29% (4)	Male 48% (10)
	Female 71% (10)	Female 52% (11)
**Age minimum – maximum (median) years**	Range 19–51 (28)	Range 22–54 (31)
**Years post BCG prime minimum – maximum (median)**	Range 7–38 (12)	Range 1–38 (18)
**Country of birth**		
**UK**	93% (13)	86% (14)
**Africa**	0%	12% (2)
**Other**	7% (1)	24% (5)
**Healthcare worker**	36% (5)	29% (6)
**Significant travel history**	21% (3)	10% (2)

All subjects completed a diary card recording local and systemic adverse events and body temperature for 7 days following vaccination. In addition, solicited adverse events were collected at each clinic visit.

### Interventions

In the BCG-BCG study, volunteers were vaccinated with BCG (a single immunisation with BCG (BCG Denmark, Statens Serum Institute, Copenhagen, Denmark), 100 µl administered intradermally over the deltoid region (n = 14)). In the BCG-MVA85A study, subjects were vaccinated intradermally with a single immunisation of 5×10^7^ pfu MVA85A [24].

### Immunogenicity Analyses

#### ELISpot Analysis

A standardised *ex vivo* IFNγ ELISpot assay was performed on PBMC taken at the following time points: at screening (prior to the Heaf test), and then at 1, 4, 8, 12, and 24 weeks after vaccination on fresh PBMC from subjects in both trial groups. Blood was also taken at 52 weeks after vaccination in those subjects still residing in the area [24]. Briefly, 300,000 fresh PBMCs per well were plated directly onto the ELISpot plate (MAIP, Millipore) in the presence of either Tuberculin Purified Protein Derivative (PPD) at 20 µg/ml, purified antigen 85 complex (10 µg/ml), or antigen 85A pooled peptides (7 pools of 9–10 15-mer peptides spanning the length of antigen 85A, which overlapped by 10 amino-acids). The final concentration of each peptide in the well was 10 µg/ml. The PBMC were incubated with antigen (PPD, antigen 85, or antigen 85A peptide pools) for 18 hours at 37°C. Streptokinase/Streptodornase and PHA were used in all assays as positive controls and cells and media alone as the negative control. Assays were performed in duplicate and the results were averaged.

The ELISpot data were analysed by subtracting the background counts (mean number of spots in the medium and cells alone control wells) from the mean number of spots in wells with antigen and cells, using an AID ELISpot 04 reader (AID Diagnostika GmbH, Strassberg, Germany). Counts of less than 5 spots/well were considered as negative. A well was considered positive if the count was at least twice that in the negative control wells and at least 5 spots more than the negative control wells. For the peptide pool wells, the results were summed across all the peptide pools (SPP) for each volunteer, at each time point. This will count twice a T cell that responds to any of the 10-mer overlap regions that occur in two pools with adjacent peptides, as each pool contains non-overlapping peptides.

An area under the curve (AUC) analysis for IFNγ ELISpot responses was performed to compare BCG - BCG and BCG - MVA85A vaccine regimens over time (up to 52 weeks).

### Dendritic Cell Mediated CD8+ T Cell Amplification

The flow cytometry assays were performed on cryopreserved PBMC obtained from subjects in both the BCG-BCG and BCG-MVA85A groups at screening (prior to the tuberculin skin test), and then at 1, 4, 8, 12 and 24 weeks after vaccination allowing comparability between groups. In this assay, PBMC were co-cultured with monocyte derived dendritic cells (DC) to enhance the detection of *ex vivo* CD8+ T cell responses.

DC were produced using the standard method of culturing with IL-4 and GMCSF [29]. At day 5 they were activated with 10 ng/ml LPS. At 16 hour post-activation, the DC were washed, peptide-pulsed for 45 minutes, and washed again. Preliminary experiments performed with DC pulsed with BCG showed no detectable CD8+ T cell response in the BCG-BCG regime and so this was not investigated further. PBMC were co-cultured in 24-well plates at a ratio of 15∶1 (PBMC∶DC) in the presence of IL-7 at 25 ng/ml on day 1, then IL-2 was added at 0.5 ng/ml on day 3, and subsequently on day 7 and day 10. The culture was washed on day 13 and peptide re-stimulation (10 µg/ml) was performed on day 14, alongside PHA and unstimulated cells. Brefeldin A was added after 4 hours and after a further 12 hours intracellular cytokine staining was performed to detect IFNγ using the Becton Dickinson intracellular cytokine staining kit with GolgiPlug™, according to the manufacturer's protocol.

An HLA-A2 specific CD8+ immunodominant antigen 85A peptide pentamer, KLIANNTRV [30] was selected as this has previously been shown to be recognised in BCG vaccinated subjects. A previously defined HLA-A2 restricted CD8+ T cell epitope was selected as HLA-A2 is the most common HLA Class I antigen and this therefore enabled us to analyse the maximum number of subjects using a single epitope, KLIANNTRV. To evaluate and quantify CD8+ T cell responses, 5 subjects in the BCG-BCG trial and 8 subjects in the BCG-MVA85A trial all of whom were HLA-A2 positive were analysed.

Tetramers for EBV (EBNA 3A, HLA-B35-restricted YPLHEQHGM) and HIV epitopes (gag; p17 HLA-A24-restricted KYRLKHLVW and p46 HLA-A6802-restricted ETAYFILKL) were used as a positive and negative control (for short term culture conditions), respectively [27], [28], [31].All antigens were used at 10 µg/ml.

Cells were incubated with KLIANNTRV pentamer labelled with APC for 60 minutes at 37°C. Cells were then cooled to 5°C and incubated with PE-conjugated CD8 mAb (Becton Dickinson) for 30 minutes, washed and incubated with FITC-conjugated IFNγ and PerCP-conjugated CD3 mAb (Becton Dickinson) for 30 minutes at 5°C.

Cells were acquired using flow cytometry on a FACS Calibur (Becton Dickinson) machine and were analyzed using FlowJo software by gating on the lymphocyte population in forward scatter (FSC) and side scatter (SSC), and gating on live cell populations. Gating was then performed for CD8^HI+^ population to determine if they were antigen specific and producing IFNγ. These gated statistics were used and the specific responses (i.e. the percentage of positive stimulated cells minus the percentage of positive unstimulated cells) were displayed in a histogram. The data shown are representative of triplicate experiments.

### Statistical Methods

A Mann-Whitney test was used for all comparisons between groups, areas under the curve and comparisons between early and late time points (week 1 and week 52) between groups, and the Binomial method was used to estimate the confidence intervals using STATA 9 [32]

## Results

### Recruitment and Demographics

Subjects were recruited from February 2004 to November 2005 for the BCG-BCG group, and from March 2003 to June 2006 for the BCG-MVA85A group. All subjects were followed up for safety and immunogenicity for 24 weeks after vaccination, with an additional visit at 52 weeks for 12 subjects from the BCG-BCG regime and 13 subjects from the BCG-MVA85A regime. There was 1 (7%) of 14 subjects in the BCG-BCG group born outside the UK, whereas 7 (33%) of 21 subjects in the BCG-MVA85A group were born outside the UK. Three (21%) of BCG-BCG subjects had significant travel history compared with 2 (10%) of BCG-MVA85A subjects.

### Safety of BCG-BCG Vaccination Compared With BCG-MVA85A Vaccination

The BCG-BCG and BCG-MVA85A regimens were well tolerated with no serious or severe vaccine related systemic adverse events. There were no marked differences in local and systemic adverse events between BCG-BCG (n = 14) and BCG-MVA85A (n = 21) vaccination regimens.

All subjects in the BCG-BCG and BCG-MVA85A regimens experienced some mild local adverse events related to the vaccination. This included redness, pain and induration that was experienced by all subjects in both regimens, with some subjects also experiencing pruritus (Table 2A).

**Table 2:** Local and Systemic Adverse Events in Subjects After the BCG-BCG or BCG-MVA85A Vaccination Regimen.

**A: Local Adverse Events**		
**Adverse event**	**BCG-BCG (n = 14)**	**BCG–MVA85A (n = 21)**
**Redness**	100% (14)	100% (21)
**Pruritus**	64% (9)	48% (10)
**Pain**	100% (14)	100% (21)
**Induration**	100% (14)	100% (21)
**B: Systemic Adverse Events**		
**Adverse event**	**BCG-BCG (n = 14)**	**BCG–MVA85A Long boosting interval (n = 21)**
**Fever**	0% (0)	5% (1)
**Feverish**	14% (2)	24% (5)
**Arthralgia**	29% (4)	14% (3)
**Headache**	36% (5)	38% (8)
**Myalgia**	36% (5)	24% (5)
**Nausea**	7% (1)	5% (1)
**Diarrhoea**	0%	0% (0)
**Vasovagal syncope**	0%	0% (0)
**Axillary LN**	14% (2)	5% (1)
**Alterations in haem/biochem**	7% (1)	0% (0)
**Infection at injection site**	7% (1)	5% (1)

All systemic adverse events reported were mild in severity. The most common systemic adverse events were headache (5 (36%) of 14 subjects receiving BCG-BCG and 8 (38%) of 21 subjects receiving BCG-MVA85A), and myalgia (5 (36%) of 14 subjects receiving BCG-BCG and 5 (24%) of 21 subjects receiving BCG-MVA85A). The adverse event profiles were comparable between the two vaccine regimens (Table 2A and B).

### BCG-MVA85A induced significantly stronger cellular immune responses to antigen 85 protein and antigen 85A SPP than BCG-BCG vaccination

**Figure 2:**
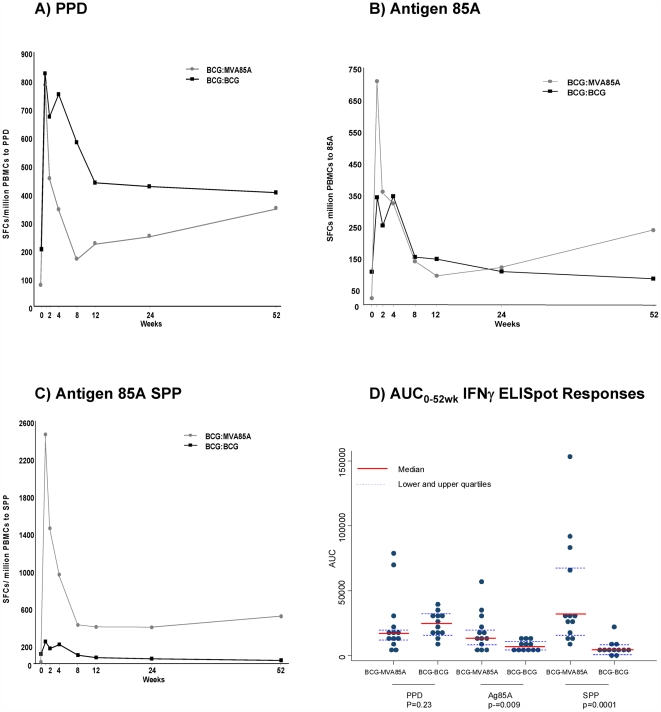
Median IFNγ ELISpot Responses to PPD, antigen 85A protein, and SPP, up to 52 Weeks Post-Vaccination. Median IFNγ ELISpot responses were assessed for PBMC from subjects in the BCG-BCG and BCG-MVA85A vaccination regimens against: (A) Tuberculin PPD; (B) Purified antigen 85 protein; (c) summed pooled peptide (SPP) of antigen 85A peptides. (d) A dot plot of AUC_0–52weeks_ values calculated for PPD, antigen 85A protein, and SPP, and statistical analyses performed using Mann-Whitney test to compare results from BCG-BCG and BCG-MVA85A subjects. The results demonstrate significantly higher responses to antigen 85A protein (p = 0.009) and SPP (p = 0.0001) in BCG-MVA subjects compared with BCG-BCG subjects.

The immune responses to PPD, antigen 85, and antigen 85A SPP were compared between subjects in the BCG-BCG and BCG-MVA85A trials in PBMC at various time points over the 52-week post-vaccination period. In the BCG-BCG subjects, a strong cellular immune response to PPD was elicited at 1-week post-vaccination, resulting in an increase in IFNγ ELISpot responses (median 824 spot forming cells (SFC)/10^6^ peripheral blood mononuclear cells (PBMC)) above levels observed at screening (median 205 SFC/10^6^ PBMC; Figure 2A). The cellular immune response was lower by 12 weeks post-vaccination (median 467 SFC/10^6^ PBMC), and reached a plateau at these levels which persisted for at least 52 weeks after vaccination (median 402 SFC/10^6^ PBMC). Similarly, in BCG-MVA85A subjects, the peak response to PPD was observed 1-week post-vaccination (median 783 SFC/10^6^ PBMC). These levels dropped at 12 weeks post-vaccination (median 223 SFC/10^6^ PBMC), increasing marginally at 52 weeks (median 347 SFC/10^6^ PBMC).

The response to antigen 85 protein in the BCG-BCG subjects was lower than the PPD response; peaking at 1 week post-vaccination (median 340 SFC/10^6^ PBMC) from screening (median 105 SFC/10^6^ PBMC). A low level response to this antigen continued to be observed throughout the 52-week post-vaccination period (Figure 2B). In contrast, there was a marked increase in response to antigen 85 protein in the BCG-MVA85A subjects at 1-week post-vaccination (median 790 SFC/10^6^ PBMC) over values observed at screening (median 27 SFC/10^6^ PBMC; Figure 2B). The response to antigen 85A declined up to 12-weeks post-vaccination (median 113 SFC/10^6^ PBMC), and response levels were marginally higher at 52 weeks (median 237 SFC/10^6^ PBMC).

In BCG-BCG subjects, the response to SPP increased at 1 week post-vaccination (median 236 SFC/10^6^ PBMC) compared with screening (median 102 SFC/10^6^ PBMC). The response was reduced to baseline levels (median 95 SFC/10^6^ PBMC) at 8-weeks post-vaccination, where this plateau continued to 52 weeks (median 34 SFC/10^6^ PBMC). In BCG-MVA85A subjects, the response to SPP increased markedly at 1 week post-vaccination (median 2147 SFC/10^6^ PBMC), compared with values at screening (median 13 SFC/10^6^ PBMC). The response to SPP declined by 12 weeks (median 390 SFC/10^6^ PBMC), and then increased marginally by 52 weeks post-vaccination (median 506 SFC/10^6^ PBMC).

To analyse the effect of the vaccine regimens over the 52-week post-vaccination period, an area under the curve (AUC) analysis of the IFNγ ELISpot responses was performed on subjects present at all time points (1, 2, 4, 8, 12, 24, and 52 weeks). The results demonstrated that the boosting effect of MVA85A (n = 13) was significantly greater than the booster effect of BCG (n = 12) in enhancing the cellular response to antigen 85A (p = 0.009) and SPP (p = 0.0001) over the 52-week period (Figure 2D and Table 3). The response to PPD was not significantly different between the BCG–BCG and BCG–MVA85A groups (Table 3).

**Table 3:** AUC_0–52weeks_ IFNγ ELISpot Responses in BCG-BCG and BCG-MVA85A Subjects.

Outcome of area under the curve 0–52 week	BCG-MVA85A		BCG-BCG		Difference in medians (95% CI) a	P-value^b^
	n	Median (IQR)	n	Median (IQR)		
**PPD**	13	16736 (11057, 26379)	12	24201 (16408, 32634)	−5603 (−15628 to 3654)	0.23
**Antigen 85A**	13	12937 (7256, 26401)	12	6193 (4915, 12216)	6446 (1078 to 14982)	0.009
**SPP**	13	31760 (15978, 74853)	12	4256 (2453, 6697)	24379 (11133 to 65216)	0.0001

IQR = inter quartile range; AUC calculated between baseline and 52 weeks post vaccination.

a estimated using the Binomial method.

b Mann-Whitney test.

### 1 Week Post-Vaccination

**Figure 3:**
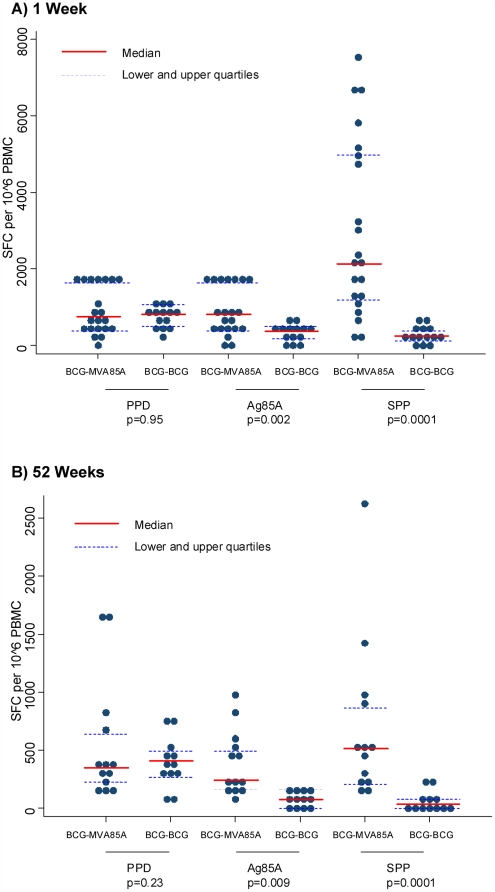
Comparison of IFNγ ELISpot Responses in BCG-BCG and BCG-MVA85A Subjects. IFNγ ELISpot responses for BCG-BCG and BCG-MVA85A vaccine regimens at (A) 1 week and (B) 52 weeks post-vaccination. Statistically significant differences between the two vaccination regimens were assessed using Mann-Whitney test. BCG-MVA85A subjects had significantly greater responses to antigen 85A protein and SPP compared with BCG-BCG subjects.

To examine the significance of the peak effect of the vaccination regimens at 1-week post-vaccination, statistical analysis of the cellular immune responses to PPD, antigen 85A protein and SPP was performed (BCG∶BCG n = 21 BCG∶MVA85A n = 14). A strong cellular immune response was observed for PPD-specific PBMC in both BCG-BCG and BCG-MVA85A subjects at 1 week after vaccination (median 707 SFC/10^6^ PBMC and 824 SFC/10^6^ PBMC, respectively), with no significant differences in response to PPD between the 2 groups (p = 0.95; Figure 3A). In contrast, at 1 week after vaccination, a significantly higher response for PBMC from BCG-MVA85A subjects was observed against antigen 85A protein (median 790 SFC/10^6^ PBMC) and SPP (median 2147 SFC/10^6^ PBMC) than in PBMC from BCG-BCG subjects (median 340 SFC/10^6^ PBMC against antigen 85A protein, p = 0.002, and median 236 SFC/10^6^ PBMC against SPP, p<0.0001; Table 4, and Figure 3A).

**Table 4:** Statistical Analysis of 1-Week and 52-Week Post-Vaccination IFNγ ELISpot Responses in BCG-BCG and BCG-MVA85A Subjects.

Outcome at 1 week post-vaccination	BCG-MVA85A		BCG-BCG		Difference in medians (95% CI)^a^	P-value^b^
	n	Median (IQR)	n	Median (IQR)		
PPD	21	707 (389, 1653)	14	824 (521, 967)	−21 (−328 to 576)	0.95
Antigen 85A	21	790 (422, 1653)	14	340 (182, 464)	427 (167 to 1090)	0.002
SPP	21	2147 (1172, 5084)	14	236 (148, 399)	1939 (1074 to 4520)	<0.0001
Outcome at 52 weeks post-vaccination						
PPD	13	347 (199, 737)	12	402 (277, 517)	−6 (−177 to 290)	0.83
Antigen 85a	13	237 (160, 555)	12	83 (20, 142)	190 (74 to 446)	0.0003
SPP	13	506 (214, 921)	12	34 (21, 73)	419 (177 to 854)	0.0001

IQR = inter quartile range.

a estimated using the Binomial method.

b Mann-Whitney test.

### 52 Weeks Post-Vaccination

To determine the level of cellular immune responses at the end of the follow-up period, 52-week post-vaccination samples were examined, (BCG∶BCG n = 12, BCG∶MVA85A n = 13). Statistical analysis of subjects at 52-weeks post-vaccination demonstrated a moderate response to PPD in PBMC from BCG-BCG subjects (median 402 SFC/10^6^ PBMC) and BCG-MVA85A subjects (median 347 SFC/10^6^ PBMC), with no significant difference. A significantly greater antigen 85A specific cellular immune response was detected in PBMC from BCG-MVA85A subjects (median 237 SFC/10^6^ PBMC to recombinant protein and median 506 SFC/10^6^ PBMC to the SPP) than in PBMC from BCG-BCG subjects (median 83 SFC/10^6^ PBMC against antigen 85A, p = 0.0003, and 34 SFC/10^6^ PBMC against SPP, p = 0.0001; Table 4 and Figure 3B).

### Dendritic cell mediated amplification of IFNγ secreting, antigen 85A peptide specific, CD8+ T cells from PBMC

**Figure 4:**
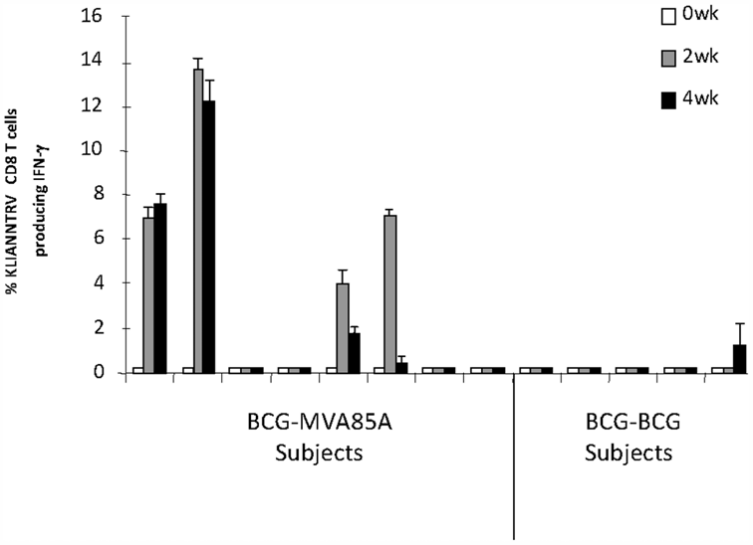
Dendritic Cell Amplification of TB-Specific, IFNγ Producing CD8 T Cells. TB-specific CD8+ T cells producing IFNγ in response to an immunodominant peptide KLIANNTRV, were identified in HLA-A2 positive subjects in the BCG-BCG and BCG-MVA85A vaccine regimens at 2 and 4 weeks. TB-specific CD8+ T cells were not detectable above 0.5% TB-specific CD8+ T cells at the 0, 1, 8, and 24 week timepoints analysed (data not shown). Values are presented as mean (+SD) and represents triplicate samples.

In the five HLA-A2+ BCG-BCG subjects studied in this analysis, only one subject had very low levels of IFNγ secreting, CD8+ T cells recognising KLIANNTRV (1.5% of total CD8 population)at one time point (4-weeks) post-vaccination (Figure 4). In contrast, in the eight HLA-A2+ BCG-MVA85A subjects, IFNγ secreting, CD8+ T cells recognising KLIANNTRV were detectable at 2- and 4-weeks post-vaccination in 4 (50%) of 8 subjects, at levels up to approximately 10-fold higher than those observed for BCG-BCG subjects (up to 14% total CD8 population).

## Discussion

In this current study, revaccination with BCG was well tolerated with no unexpected or serious adverse events up to 52 weeks post-vaccination. This was similar to the adverse event profile observed in subjects in a previously reported trial using the BCG-MVA85A vaccine regimen [24].

Vaccination with BCG has variable efficacy, failing to protect against pulmonary disease in adults, whilst affording protection in childhood [4]. The most widely accepted explanation for the variable efficacy of BCG in many global trials is that prior exposure to environmental mycobacteria either inhibits the replication and development of BCG induced protective immune response or masks the effect of BCG vaccination by providing a similar degree of anti-mycobacterial immunity [11], [33]. In this current study, the differences in cellular immune responses between these two vaccine regimens could not be explained by possible differences in the levels of mycobacterial antigen exposure at baseline. To confirm there was no difference between mycobacterial antigen exposure between trials, the trial inclusion criteria, including baseline Tuberculin skin test responses, were the same for both trials.

The results from this study provide evidence that there are elevated cellular immune responses to *M.tb* antigens following BCG-BCG and BCG-MVA85A vaccine regimens. The IFNγ ELISpot response to PPD in BCG-BCG subjects was high, and similar to BCG-MVA85A subjects at both early (week 1) and late (week 52) time points, and over the 52 weeks period (as determined by AUC values), with no significant differences observed between the two vaccine regimes. Boosting BCG with BCG may result in boosting of many of the different secreted antigens within PPD, whereas boosting with MVA85A will only result in boosting of the antigen 85A component of BCG.

In contrast to the PPD responses, in this study, we have shown that subjects vaccinated with MVA85A have significantly higher cellular immune responses to antigen 85A protein and antigen 85A SPP at peak (week 1) and plateau (week 52) time points, and over the 52 week period (as determined by AUC values) than in BCG-BCG subjects. Studies have demonstrated that reactivity against antigen 85A [19] and antigen 85B protein [34], invoke a protective immunity to TB. Immunization with a vaccine construct that encodes antigen 85A confers protection against challenge with live TB in small animals [21], [22], [23].

Although significant differences were observed using the *ex vivo* IFNγ ELISpot assays, this assay only detected CD4+ T cell responses. No CD8+ T cell responses have been identified before this current study. Therefore, we used DC to amplify the low numbers of CD8+ T cells that were otherwise not detectable, and compared between boosting regimens. An HLA-A2 restricted immunodominant peptide (KLIANNTRV) from antigen 85A was selected to identify MHC class I-restricted, CD8+ T cells. KLIANNTRV has been shown to be recognised by PBMC stimulated with BCG in 50% of BCG-vaccinated subjects [30]. Following amplification of CD8+ T cells using DC, we observed that revaccination with BCG induced detectable, but low frequency of pentamer (KLIANNTRV)+, IFNγ+, CD8+ T cells at a single time point in only one of 5 HLA-A2+ subjects. The magnitude (up to 10-fold higher) of CD8+ T cell recognition of the immunodominant KLIANNTRV peptide was higher in the BCG-MVA85A subjects, was detectable in 4 of 8 subjects and detectable at 2 different time points (in 50% of subjects). It is clear that a higher frequency of CD8+ T cells can be detected in the BCG-MVA85A regimen than in the BCG-BCG regimen using this highly sensitive assay. The clinical significance of this enhanced CD8+ T cell response can only be determined in large scale efficacy trials. It is possible that the levels of antigen-specific CD8+ T cell responses remain below the limit of detection in BCG-BCG subjects, but given the increased sensitivity of the DC-mediated amplification method used in this study, this would imply they are at very low frequency indeed. The results of this study demonstrate that DC are a useful and powerful tool in providing the stimulation required to amplify the low frequency CD8+ T cells in PBMC samples to detectable levels.

CD8+ T cell responses to mycobacteria have been shown to be induced through vaccination with BCG in older subjects, or following latent infection or active disease [30], [35], [36], [37]. However, the widely held view is that BCG vaccination results in suboptimal CD8+ T cell responses. Although CD4+ T cells are the predominant cells controlling TB infection, there is evidence in animal studies that CD8+ T cells are involved in protection against TB [23], [38], [39]. Protection against *M. tb*, an intracellular pathogen, probably requires a finely balanced interplay between cells of the innate and adaptive immune system. CD8+ T cell responses may play role in the initial control of TB infection and this may be elicited by presentation from neutrophils during the initial innate immune response [40], [41] or through interactions with neutrophils and DC [42]. Neutrophils have been shown to cross-present pathogen-derived and soluble antigens to CD8+ T cells *in vivo* [42]. This may go some way to explain the differences observed in whole blood assays where neutrophils are present and may initiate CD8+ T cell proliferation, compared with PBMC preparations where neutrophils are not present. Indeed, it has been shown that CD8+ T cells can be detected by flow cytometry in whole blood samples from adults [36], [37] and infants [43], [44] vaccinated with BCG. Although pentamer (KLIANNTRV)+, IFNγ+, CD8+ T cells could be detected in the BCG-BCG and BCG-MVA85A subjects in our study, it is possible that other, functional, CD8+ T cells exist as we have shown in subjects who received BCG-MVA85A [45]. As these cells occur at low frequency, it may be possible that the use of whole blood samples coupled with DC amplification could be used as a powerful tool for amplifying low frequency CD8+ T cells to asses their functionality.

In summary, the results in this study have demonstrated that in the BCG-MVA85A vaccination regimen, significantly stronger cellular immune responses to antigen 85A can be induced compared with revaccination with BCG-BCG. IFNγ is clearly an important cytokine involved in protection against TB and has been used as a measure of vaccine “take” [12] in Phase I BCG-MVA85A clinical trials [24], [45], [46], [47]. As further research is performed, it is highly likely that other correlates of protection will emerge. Proofs of concept efficacy trials are due to start in early 2009, and will enable us to evaluate whether enhanced immune responses following BCG-MVA85A result in improved protection.

## Supporting Information

Protocol S1 Trial protocol
(0.12 MB PDF)

Checklist S1 CONSORT Checklist
(0.05 MB DOC)
