# Correction: Safety and Immunogenicity of Boosting BCG Vaccinated Subjects with BCG: Comparison with Boosting with a New TB Vaccine, MVA85A

**DOI:** 10.1371/annotation/ec8cc565-bf24-4898-b7ba-6e0423d5809f

**Published:** 2011-02-25

**Authors:** Kathryn T. Whelan, Ansar A. Pathan, Clare R. Sander, Helen A. Fletcher, Ian Poulton, Nicola C. Alder, Adrian V. S. Hill, Helen McShane

The following information was missing from the Funding section: "The study was supported by funding from the NIHR Oxford Biomedical Research Centre programme."

